# FISHFactor: a probabilistic factor model for spatial transcriptomics data with subcellular resolution

**DOI:** 10.1093/bioinformatics/btad183

**Published:** 2023-04-11

**Authors:** Florin C Walter, Oliver Stegle, Britta Velten

**Affiliations:** Division of Computational Genomics and System Genetics, German Cancer Research Center (DKFZ), Heidelberg 69120, Germany; Genome Biology Unit, European Molecular Biology Laboratory (EMBL), Heidelberg 69117, Germany; Division of Computational Genomics and System Genetics, German Cancer Research Center (DKFZ), Heidelberg 69120, Germany; Genome Biology Unit, European Molecular Biology Laboratory (EMBL), Heidelberg 69117, Germany; Cellular Genetics Programme, Wellcome Sanger Institute, Wellcome Genome Campus, Hinxton, Cambridge CB10 1SA, United Kingdom; Division of Computational Genomics and System Genetics, German Cancer Research Center (DKFZ), Heidelberg 69120, Germany; Cellular Genetics Programme, Wellcome Sanger Institute, Wellcome Genome Campus, Hinxton, Cambridge CB10 1SA, United Kingdom

## Abstract

**Motivation:**

Factor analysis is a widely used tool for unsupervised dimensionality reduction of high-throughput datasets in molecular biology, with recently proposed extensions designed specifically for spatial transcriptomics data. However, these methods expect (count) matrices as data input and are therefore not directly applicable to single molecule resolution data, which are in the form of coordinate lists annotated with genes and provide insight into subcellular spatial expression patterns. To address this, we here propose FISHFactor, a probabilistic factor model that combines the benefits of spatial, non-negative factor analysis with a Poisson point process likelihood to explicitly model and account for the nature of single molecule resolution data. In addition, FISHFactor shares information across a potentially large number of cells in a common weight matrix, allowing consistent interpretation of factors across cells and yielding improved latent variable estimates.

**Results:**

We compare FISHFactor to existing methods that rely on aggregating information through spatial binning and cannot combine information from multiple cells and show that our method leads to more accurate results on simulated data. We show that our method is scalable and can be readily applied to large datasets. Finally, we demonstrate on a real dataset that FISHFactor is able to identify major subcellular expression patterns and spatial gene clusters in a data-driven manner.

**Availability and implementation:**

The model implementation, data simulation and experiment scripts are available under https://www.github.com/bioFAM/FISHFactor.

## 1 Introduction

Transcriptomic profiling of individual cells using single-cell RNA sequencing is now a widely accessible tool for studying cellular heterogeneity in tissues and has contributed to the discovery of new cell types. However, single-cell RNA sequencing protocols are based on a disassociation step and therefore can provide only limited insight into the spatial organization of tissue and no information at all about the localization of RNA molecules within a cell. To address this, a growing number of spatially resolved transcriptomic technologies are being developed that allow measurements of gene expression while retaining spatial context (Rao et al. 2021, Palla et al. 2022). For example, next-generation sequencing coupled with spatial barcodes provides whole transcriptome measurements of tissue regions (Ståhl et al. 2016, Rodriques et al. 2019), but the resolution of current methods is at most at the level of individual cells and cannot resolve subcellular patterns. On the other hand, imaging-based techniques such as *in situ* sequencing (Ke et al. 2013, Lee et al. 2015, Chen et al. 2018, Wang et al. 2018) or fluorescence in situ hybridization (FISH) achieve subcellular resolution by measuring spatial positions of individual molecules. While FISH technologies were originally limited to the detection of a single or at most a handful of genes (Femino et al. 1998, Raj et al. 2008, Lyubimova et al. 2013), advances in imaging technologies, sequential hybridization, and barcoding strategies nowadays enable probing tens to thousands of genes in a single experiment (Lubeck and Cai 2012, Lubeck et al. 2014, Chen et al. 2015, Eng et al. 2017, 2019, Codeluppi et al. 2018), thus rendering such techniques increasingly applicable for the identification of subcellular gene expression patterns at scale.

Despite the availability of technologies that provide single-molecule resolution, most established analysis strategies for processing these data do not fully exploit the given resolution. Instead, RNA quantifications are limited to cellular resolution, for example, by aggregating the numbers of molecules per cell (Chen et al. 2015, Codeluppi et al. 2018, Eng et al. 2019), or used only for the task of cell type inference or clustering (Qian et al. 2020, Littman et al. 2021, Park et al. 2021, Partel and Wählby 2021). Thereby, such approaches cannot model subcellular gene expression patterns, which can provide important insights into cellular states, heterogeneity within cell types (Buxbaum et al. 2015, Xia et al. 2019) and can modulate the function of genes (Eng et al. 2019). A recently developed tool to explicitly analyse subcellular gene expression patterns is Bento (Mah et al. 2022). This tool computes spatial statistics of RNA expression and cell morphology and provides visualization tools to perform exploratory analyses. Moreover, it includes a classification model for the subcellular localization of individual genes. However, it requires the allowed spatial patterns to be predefined and therefore is only of limited usefulness for de novo discovery. With the increasing throughput of single-molecule techniques, it will become ever more important to identify major subcellular gene expression patterns in a data driven manner and use them as additional source of information when dissecting cell-to-cell heterogeneity.

Factor models are already widely used for the unsupervised discovery of the principal sources of variation in high-dimensional molecular datasets (Brunet et al. 2004, Witten et al. 2009, Argelaguet et al. 2018, 2020, Risso et al. 2018, Stein-O’Brien et al. 2018), and recent extensions to spatial data have successfully identified spatial gene expression patterns at the *cellular* level (Berglund et al. 2018, Velten et al. 2022, Townes and Engelhardt 2023). However, these methods cannot leverage the *subcellular* resolution of spatial transcriptomics data, as they require a count matrix as input and consequently are not directly applicable to single molecule resolved data, which are lists of coordinates annotated with gene labels. To apply these methods, it is currently required to crudely aggregate the data, using spatial binning, or summation of molecules per cell, which involves additional parameters and results in a loss of the exact spatial information.

**Figure 1:**
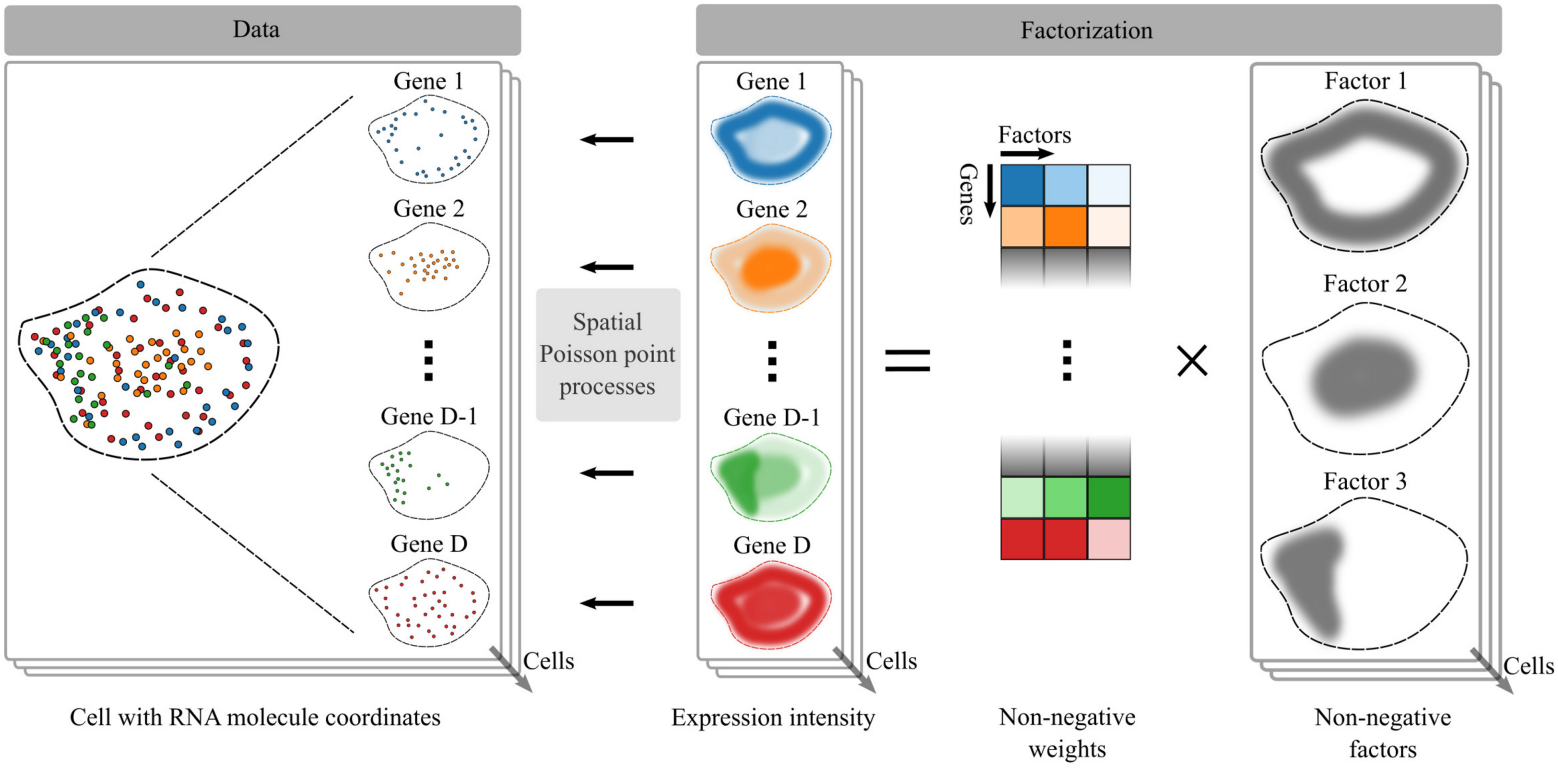
Illustration of FISHFactor for the analysis of spatial transcriptomics data with subcellular resolution. The input dataset (left) consists of RNA molecule coordinates in a single or optionally multiple segmented cells, e.g. from multiplexed FISH measurements. FISHFactor models the observed coordinates as realizations of a spatial Poisson point process with a gene-wise intensity function in each cell. The intensity functions of all genes and cells are governed by a low-dimensional decomposition into non-negative spatially aware factors and non-negative weights (right, illustrated for three latent factors). Weights are shared between cells, whereas factors are specific to individual cells

To address these shortcomings, we here propose FISHFactor, a principled factor analysis framework that opens up the application of factor models for spatially resolved single-molecule data and enables the unbiased identification and discovery of subcellular expression patterns (Fig. 1). Other than existing spatial factor models, FISHFactor employs spatial Poisson point processes as observation model to explicitly model the subcellular coordinates of each RNA molecule. It can thereby fully leverage the single-molecule resolution of the data. We combine this with a spatially aware inference of factors using Gaussian processes (GPs) tailored to spatial transcriptomics data and impose interpretable factors and weights using non-negativity constraints. To enable the integration and comparison of subcellular localization patterns across a population of cells, FISHFactor jointly models the information from multiple cells in a scalable manner by inferring a shared weight matrix, while retaining independent sets of factors. We assess the model using simulated data, where we demonstrate advantages of FISHFactor over existing approaches that require spatial binning and show the benefit of jointly modeling multiple cells. We show that FISHFactor scales to very large datasets of more than 1000 cells and that it generates reproducible results. Using a real dataset, we illustrate the use of FISHFactor to reveal subcellular localization patterns of genes and to analyse the co-localization of genes within a cell. Moreover, we show that it is possible to train the model on a subset of cells and project the remaining data on latent factors using the trained model.

## 2 Materials and methods

### 2.1 Factor analysis for count data

Factor analysis is a dimensionality reduction technique commonly used for unsupervised analysis of high-dimensional omics datasets (Stein-O’Brien et al. 2018). Based on correlation structures in a high-dimensional feature space, the method aims to find a low-dimensional embedding in terms of a small number of latent factors, representing the major axes of variation in the data. Starting from a high-dimensional dataset $Y\in R^{N\times D}$ with *N* observations of *D* features, factor analysis finds a factorization $Y=ZW^{T}+E$ with *K* latent factors $Z\in R^{N\times K}$ (typically K≪D), associated factor weights $W\in R^{D\times K}$ and residual noise $E\in R^{N\times D}$. In contrast to nonlinear dimensionality reduction methods, factor analysis identifies latent embeddings that can be directly interpreted, because the weights linearly link each latent factor to molecular features. Formulated in a probabilistic framework, factor analysis further allows for the incorporation of prior knowledge and various sparsity assumptions through the use of appropriate prior distributions, provides uncertainty estimates for the inferred variables and can account for different data types through the use of appropriate likelihood models. As a baseline model for sequencing data we here consider a Poisson likelihood, which can account for the count nature of the data and has been successfully applied to transcriptomics data (Townes et al. 2019). The decomposition in a Poisson factor model is given by where *g* denotes a positive inverse-link function such as the exponential or softplus (Dugas et al. 2000), defined as $\text{softplus}(x)=\text{log}(1+e^{x})$.


(1) $$p(y_{nd}|W,Z)=\text{Poisson}(\lambda_{nd})$$

(2) $$\lambda_{nd}=g(\sum_{k=1}^{K}w_{dk}z_{nk}),$$

### 2.2 Non-negative factor analysis

To improve the interpretability and identifiability of factor analysis, different sparsity assumptions on the factors and weights have been employed, including sparsity on the level of features or sets of features (Witten et al. 2009, Argelaguet et al. 2018, 2020) as well as non-negativity constraints (Lee and Seung 1999, Townes and Engelhardt 2023). The latter have been found particularly useful, as they allow to find additive non-negative spatial patterns and molecular signatures. In practice, non-negativity is achieved by constraining weights and factors to non-negative values, for example, using non-negative parametrization or non-negative priors. In such a model, with Gaussian priors on the unconstrained latent variables, $\lambda_{nd}$ in Equation (1) is given by


(3) $$\lambda_{nd}=\sum_{k=1}^{K}w_{dk}z_{nk}$$

(4) $$w_{dk}=g(q_{dk})$$

(5) $$z_{nk}=g(f_{nk})$$

(6) $$q_{dk}∼N(\mu_{q},\sigma_{q}^{2})$$

(7) $$f_{nk}∼N(\mu_{f},\sigma_{f}^{2}).$$

Equations (6) and (7) represent prior distributions on the unconstrained weights and factors, respectively, where $\mu_{q}$, $\mu_{f}$, $\sigma_{q}^{2}$, and $\sigma_{f}^{2}$ are constant mean and variance parameters of a Gaussian distribution. The weights and factors are constrained to non-negative values by applying a positive inverse-link function *g* in Equations (4) and (5). Equation (3) determines the rate parameter $\lambda_{nd}$ of the Poisson distribution for the likelihood term in Equation (1) as the matrix product of non-negative weights and factors.

### 2.3 GP factor analysis

A limitation of classical factor models in applications to spatial data is the assumption of independent observations n=1,…,N. While this assumption may be appropriate for some data types, it generally does not hold for spatial data, where each observation comes with a spatial coordinate and spatial structures are present between samples. For example, gene expression profiles at nearby points are expected to be more similar than at points that are far apart. This spatial covariance can be incorporated into factor analysis by replacing the univariate Gaussian priors on factors in Equation (7) by multivariate priors that can model covariation across samples. A flexible choice for this purpose is GP priors, which provide a nonparametric framework to model continuous dependencies between samples. This has given rise to *GP factor analysis (GPFA)* (Yu et al. 2008), where independent GP priors are placed on the factors to model smooth temporal patterns. The same concept has recently been applied for the identification of patterns in spatial transcriptomics data, in combination with different likelihood models and sparsity constraints (Velten et al. 2022, Townes and Engelhardt 2023). In particular, this approach corresponds to replacing the factor prior in Equation (7) with a GP prior:


(8) $$f_{nk}=f_{k}(c_{n})$$

(9) $$f_{k}∼\text{GP}(\mu_{k},\kappa_{k}).$$

Here, $c_{n}\in R^{2}$ is the spatial coordinate of sample *n*, $\mu_{k}$ is a mean function in $R^{2}$, and $\kappa_{k}$ is a kernel function in $R^{2}\times R^{2}$. The choice of the kernel function determines the covariance structure. For example, a squared exponential kernel generates very smooth patterns, whereas a Matérn kernel leads to a more angular appearance as often observed for spatial expression patterns (Townes and Engelhardt 2023).

### 2.4 Poisson point process likelihood

In contrast to (spatial) transcriptomics datasets at the cellular level, single-molecule resolved data consist of a list of *N* coordinate vectors ${\left\{c_{n}\right\}}_{n=1,\ldots ,N}\;c_{n}\in R^{2}$ with gene annotations. For such data, the Poisson likelihood used in the discussed models can only be employed after a pre-processing step that aggregates the number of molecules in a certain spatial region or cell and ignores the exact spatial information. A more suitable likelihood model for single-molecule resolved data are Poisson point processes, which directly model the coordinates of each molecule. Poisson point processes have already been successfully used in GPFA with temporal data in neuroscience (Duncker and Sahani 2018) and for cell typing in spatial data (Qian et al. 2020) but so far have not been considered in factor models for spatial transcriptomics data. Formally, an inhomogeneous spatial Poisson point process is characterized by a non-negative intensity function $\lambda :R^{2}\to R_{\geq 0}$. A set of point coordinates then has the probability density


(10) $$p({\left\{c_{n}\right\}}_{n=1,\ldots ,N})=\text{exp}\;\left(-\int \lambda (c)\;dc\right)\prod_{n=1}^{N}\lambda (c_{n}).$$

Intuitively, this means that more points are expected in regions where λ is high, and vice versa.

### 2.5 The FISHFactor model

FISHFactor is a probabilistic factor model for single-molecule resolved spatial transcriptomics data that combines the concepts discussed in the preceding sections (Fig. 1): (i) spatially aware inference of factors using GPs, (ii) interpretable factors and weights using non-negativity constraints, and (iii) a likelihood model accounting for the nature of single-molecule data using inhomogeneous Poisson point processes. In addition, FISHFactor allows to integrate and compare inferred patterns across multiple cells by inferring a shared weight matrix.

The input data to FISHFactor consist of a list of spatial molecule coordinates ${\left\{c_{n}^{dm}\right\}}_{n=1,\ldots ,N_{dm}}\;c_{n}^{dm}\in R^{2}$ for a set of genes d=1,…,D and cells m=1,…,M. The assignment of molecules to cells is assumed to be known and can be defined from the image using existing segmentation techniques (Littman et al. 2021, Petukhov et al. 2022). FISHFactor models the coordinates as realizations of spatial Poisson point processes, where the gene- and cell-wise intensity functions $\lambda_{dm}$ are given by a decomposition into a user-defined number *K* of cell-specific factors and a weight matrix that is shared between cells. The generative model of FISHFactor is defined as


(11) $$\begin{array}{c} p({\left\{c_{n}^{dm}\right\}}_{n=1,\ldots ,N_{dm}}|\lambda_{dm})=\\ \;\text{exp}\;\left(-\int \mu_{dm}\lambda_{dm}(c)dc\right)\prod_{n=1}^{N_{dm}}\mu_{dm}\lambda_{dm}(c_{n}^{dm}) \end{array}$$

(12) $$\lambda_{dm}(c)=\sum_{k=1}^{K}w_{dk}z_{mk}(c)$$

(13) $$w_{dk}=\text{softplus}(q_{dk})$$

(14) $$z_{mk}(c)=\text{softplus}(f_{mk}(c))$$

(15) $$q_{dk}∼N(0,1)$$

(16) $$f_{mk}∼\text{GP}(0,\kappa_{mk}(s_{mk},\ell_{mk})).$$

**Figure 2:**
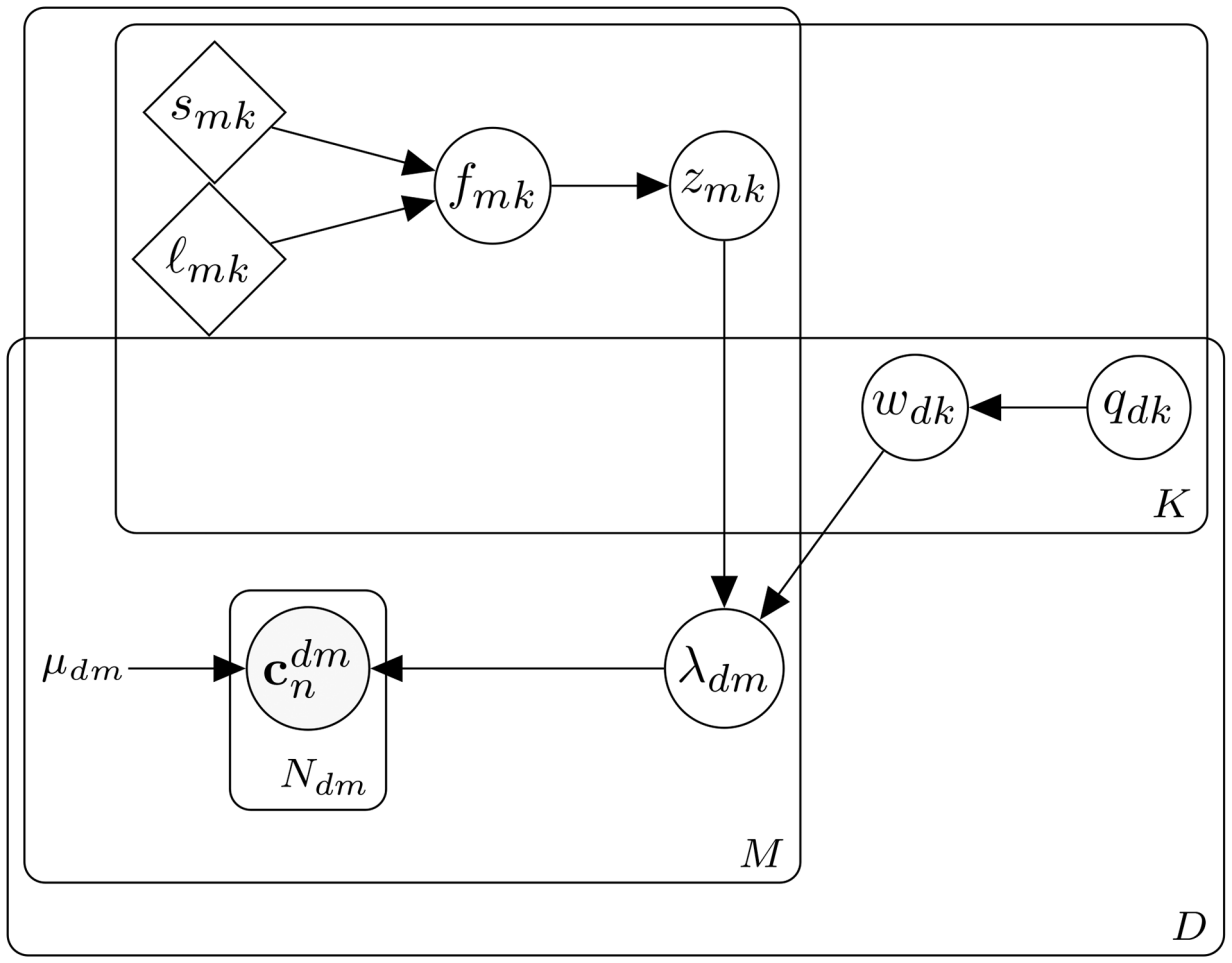
Graphical model of FISHFactor with *K* factors, *D* genes, *M* cells, and $N_{dm}$ molecules per gene and cell. Gray nodes indicate observed variables, white nodes latent variables, rhombuses learnable parameters, and $\mu_{dm}$ is a constant determined by the data

The integration limits in Equation (11) are given by the respective cell boundaries, which are estimated by thresholding a kernel density estimate based on all associated molecules. The average intensity per gene and cell $\mu_{dm}$ serves as a scale factor for the intensity function $\lambda_{dm}$ to account for differences in the overall expression intensities of genes and cells, to ensure that the inferred latent variables do not reflect abundances but subcellular patterns. It is determined as the number of molecules $N_{dm}$ divided by the cell area. The GP prior in Equation (16) uses a Matérn kernel $\kappa_{mk}$ with smoothness parameter ν and learnable output and length scales $s_{mk}$ and $\ell_{mk}$. The graphical model of FISHFactor is shown in Fig. 2.

### 2.6 Implementation

To infer the model’s latent variables in a scalable manner, FISHFactor is implemented using stochastic variational inference (Hoffman et al. 2013) and sparse approximations of the GPs (Hensman et al. 2015). In addition, a sequential update of cell-wise parameters is used to keep memory requirements constant in the number of cells (Supplementary Fig. S2), which otherwise can be a major bottleneck to the application of models to many cells. For this, every epoch consists of as many optimization steps as there are cells, whereby in every step one cell is loaded into memory, its parameters are optimized and the global weight matrix is updated. Optimization of the evidence lower bound (ELBO) is performed with a adam optimizer (Kingma and Ba 2014) with gradient clipping to increase numerical stability and a learning rate of 5 × 10^−3^. To determine convergence, the ELBO is monitored for each cell and the optimization is terminated as soon as it does not increase by a given value for any of the cells in a given number of epochs. FISHFactor is implemented using the probabilistic programming language Pyro (Bingham et al. 2019) and the low-level Pyro interface of GPyTorch (Gardner et al. 2018).

## 3 Results

All FISHFactor models in the following sections were trained on a NVIDIA Titan RTX gpu with 24 GB of NVRAM.

### 3.1 FISHFactor outperforms existing factor models on simulated data

First, we validated FISHFactor’s ability to infer subcellular expression patterns on simulated data for individual cells (*M* = 1) and compared its performance to related existing factor model implementations. We considered non-negative matrix factorization (NMF) as implemented in the *scikit-learn* package (Pedregosa et al. 2011), a widely used method for a non-negative decomposition without spatial awareness, and non-negative spatial factorization (NSF) (Townes and Engelhardt 2023), a recently proposed GP factor model for a non-negative decomposition with spatial awareness and a Poisson observation model. In contrast to FISHFactor, both methods require aggregation of molecule coordinates in spatial bins to obtain count matrices, for which we included different binning resolutions in the comparison (5 × 5, 10 × 10, 20 × 20, 30 × 30, and 40 × 40).

Data were simulated in form of molecule coordinates for 20 cells, where for each cell we independently simulated subcellular expression patterns for 50 genes using 3 latent spatial factors and corresponding gene weights and then sampled molecule coordinates from the resulting intensity function according to spatial Poisson point processes by thinning (Lewis and Shedler 1979). Fifty spatial factors were hand painted as 50 × 50 pixels grayscale images with intensity values from 0 to 1 (Supplementary Fig. S1). For every cell, three factors were randomly selected and random rotations of 0°, 90°, 180°, or 270° as well as random horizontal and vertical flips were applied. Weights were generated from a standard normal distribution, followed by a softplus transformation to positive values, multiplication with independent Bernoulli variables (*P* = .7) to induce sparsity, and normalization to a total weight of 1 for every gene. The intensity function was obtained as the matrix product of factors and weights. To examine the effect of varying molecule abundance in the data, e.g. caused by differences in detection efficiency or biological differences, we repeated the simulation with different scale factors for the intensity function (μ_*dm*_ = 50, 100, 200, 300, 400), resulting in an average of 19, 38, 77, 116, and 154 molecules, respectively, per gene and cell.

**Figure 3:**
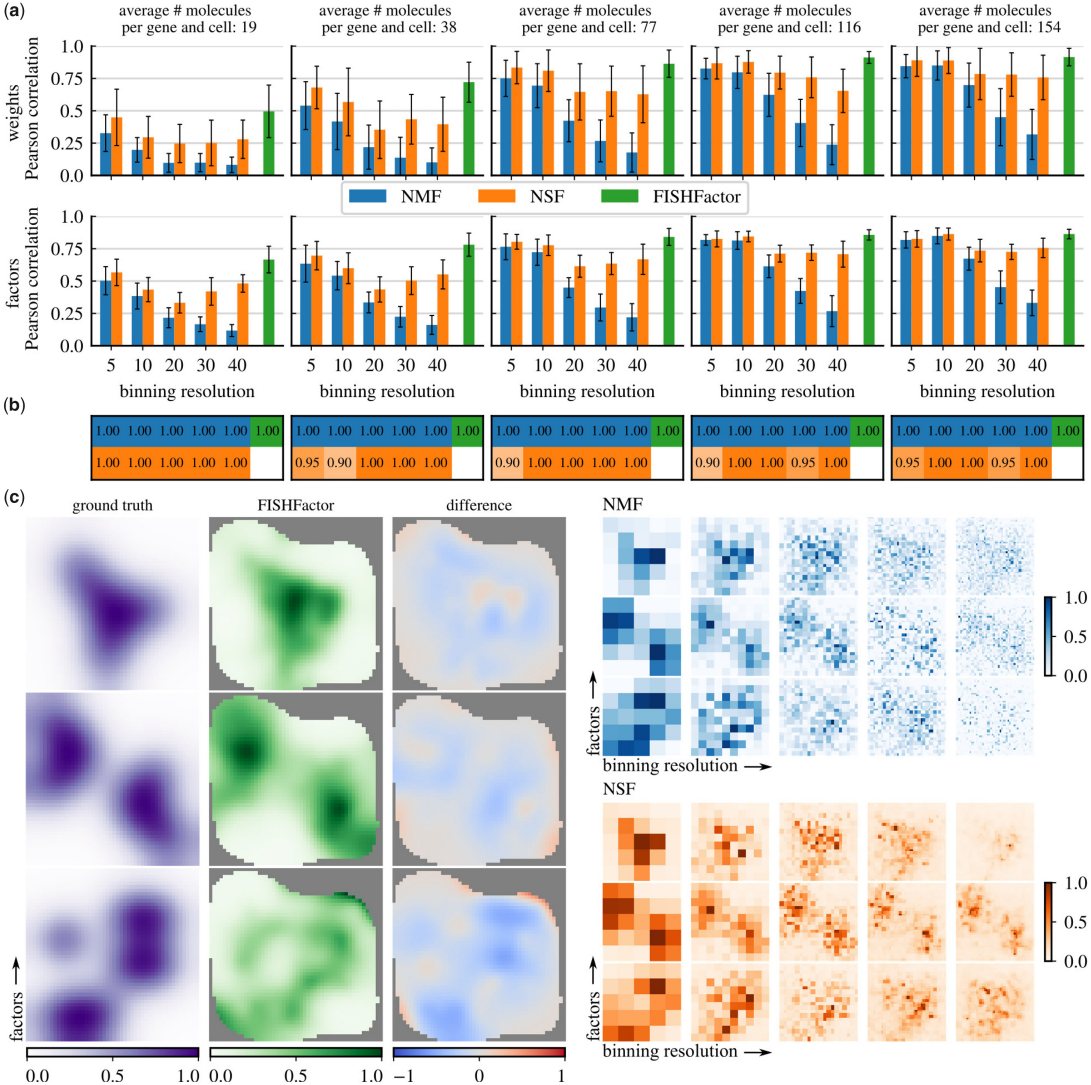
Comparison of FISHFactor to NMF and NSF at different binning resolutions on 20 simulated cells with 5 different intensity scale factors. (a) Reconstruction accuracy of the simulated weights (first row) and factors (second row). Barplots show the mean Pearson correlation across the 20 cells for FISHFactor and NMF and all cells with convergence for NSF (see b). Error bars indicate 1 SD of the mean. (b) Fractions of included cells (out of 20) for every intensity scale factor and binning resolution. NSF did not converge on all cells. (c) Exemplary visualization of ground truth and inferred factors in a single cell with an average of 68 molecules per gene. In addition, the difference between the ground truth factors and the factors inferred by FISHFactor is shown

As a postprocessing step, we normalized inferred factors to a maximum value of 1 per factor and cell, and the inferred weights to a maximum value of 1 per factor. Across all simulation scenarios, FISHFactor shows a good recovery of the simulated factors and weights (measured using Pearson correlation between simulated and inferred values, Fig. 3a), with increasing accuracy for datasets with higher number of molecules. In comparison to NMF and NSF, FISHFactor achieves a better or comparable weight and factor correlation in all scenarios. Moreover, NMF and NSF show a strong sensitivity to the choice of binning resolution, which needs to be selected in an optimal manner to reach the accuracy of FISHFactor. Such a choice can be difficult to make on real data, where no ground truth is available, and is not required in FISHFactor. At the same time, FISHFactor provides a more accurate weight reconstruction than NMF and NSF at higher spatial resolutions of factors, while for NMF and NSF accuracy in the weight reconstruction comes at the cost of a lower resolution (Fig. 3a, illustrated for a single cell with on average 68 molecules per gene in Fig. 3c). Notably, NSF fails to converge in some scenarios (fraction of cells with convergence, Fig. 3b).

### 3.2 Joint modeling of cells improves reconstruction of weights and factors

**Figure 4:**
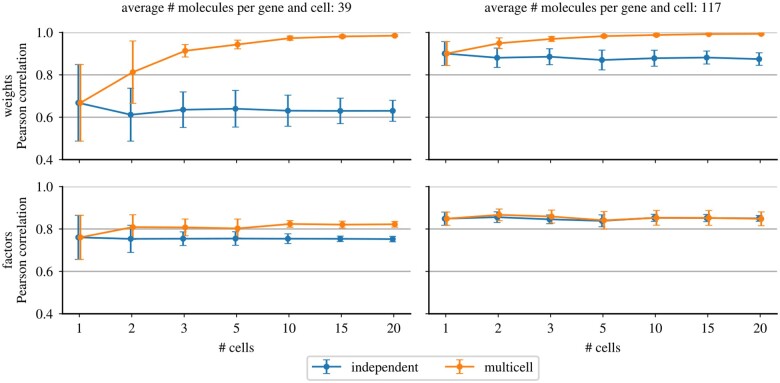
Pearson’s correlation of inferred and simulated weights (first row) and factors (second row) in 10 simulated datasets of 20 cells with shared weight matrices. In every dataset, a given number of cells (*x*-axis) were modeled using FISHFactor on all cells jointly (multi-cell, orange) or by applying FISHFactor to individual cells and averaging the results (independent, blue). Error bars show 1 SD of the mean across the 10 datasets. The first column shows results on a dataset with an average molecule count per cell and gene of 39 ($\mu_{dm}=100$), and the second column with an average count of 117 ($\mu_{dm}=300$)

In a second experiment, we investigated whether for related cells the ability of FISHFactor to share information across cells by jointly modeling their subcellular patterns benefits the reconstruction of weights and factors. For this, we simulated 10 datasets with 20 cells each as described in Section 3.1, but using a single shared weight matrix for all 20 cells, and applied FISHFactor to this data separately for each cell or jointly modeling multiple cells. We repeated this experiment with two different simulation intensity scale factors (*μ_dm_* = 100, 300), leading to an average of 39 and 117 molecules, respectively, per gene and cell. We found that the inclusion of multiple cells in the model significantly improves the reconstruction accuracy of the shared weights and, on the data with *μ_dm_* = 100, the accuracy of the inferred cell-wise factors (Fig. 4). The improvement is particularly large for the data with *μ_dm_* = 100 (Fig. 4, first column) because the smaller number of molecules makes it more difficult to derive the correct values from just one cell, and the model therefore benefits greatly from modeling multiple cells simultaneously.

### 3.3 Scalability and reproducibility

In this experiment, we investigated how the run time and memory allocation of FISHFactor scale with respect to the different model dimensions. We generated simulated data as described in Section 3.1 and set M=1 cell, D=50 genes, K=3 latent factors, and an intensity scale factor of $\mu_{dm}=100$ as the base configuration. Using this configuration, we applied FISHFactor to the simulated data and varied (i) the number of molecules per cell using intensity scales of *μ_dm_* = 50, 100, 150, 200, 250, 300, 350, 400; (ii) the number of latent factors *K* = 2, 3, 4, 5, 6, 7; and (iii) the number of jointly modeled cells *M* = 10, 20, 30, 40, 50. We generated 10 independent datasets for each scenario. Our findings indicate that both run time and memory allocation scale linearly with the number of molecules and the number of factors (Supplementary Fig. S2). As for the number of cells, the run time scales linearly while the memory allocation remains approximately constant. This is because the cells are not loaded into memory at the same time but sequentially, and the maximum memory requirement depends only on the maximum number of molecules in a single cell. However, there is a slight increase in memory allocation with the number of cells, which is expected due to the higher likelihood of including a single cell with a larger molecule count.

In order to demonstrate the feasibility of using FISHFactor with very large datasets, we generated simulated data consisting of *M* = 1000 cells with *D* = 100 genes and shared weights. We set the number of latent factors to *K* = 3, with an intensity scale factor of *μ_dm_* = 100, resulting in an average of 39 molecules per gene and cell. FISHFactor required 24.77 h for training to converge and allocated a maximum of 8.45 GB of memory. The average correlation of inferred and simulated values was *R* = 0.996 for the weights and *R* = 0.852 for the factors (Supplementary Fig. S3).

To evaluate the impact of using different random seeds on the reproducibility of inferred weights and factors, we ran FISHFactor with 10 different random seeds on five datasets (intensity scale factors $\mu_{dm}=50,100,200,300,400$). Each dataset consisted of 20 cells with shared weight matrices. Our findings show that different random seeds produce reproducible results for the inferred parameters, with an average and minimum correlation of 0.999 and 0.999 for the weights and 0.976 and 0.958 for the factors, respectively (Supplementary Fig. S4).

### 3.4 FISHFactor reveals major gene clusters and subcellular expression patterns in 3T3 cells

**Figure 5:**
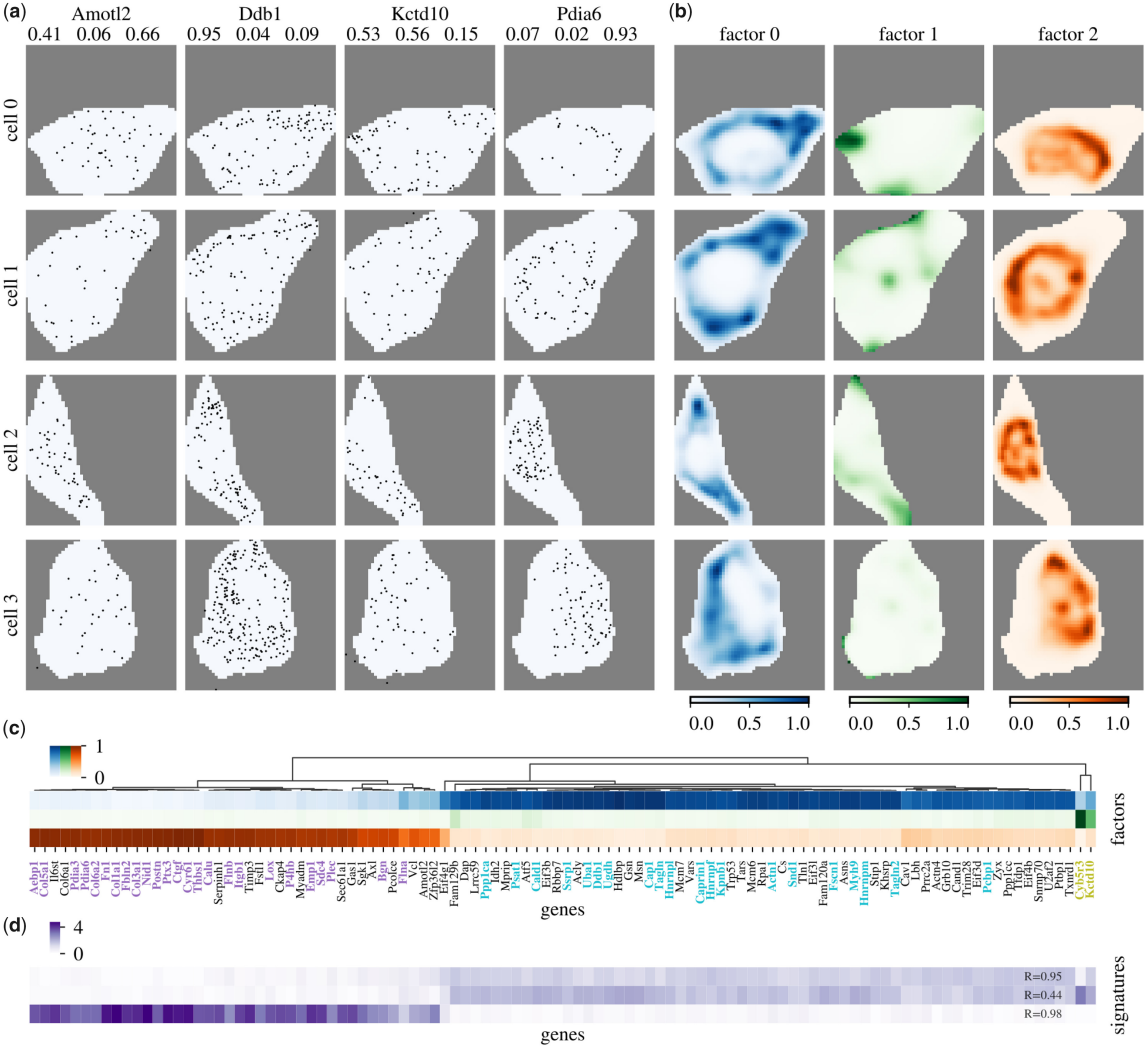
Application of FISHFactor to a dataset from cultured mouse embryonic fibroblasts (NIH/3T3) (Eng et al. 2019). (a) Molecule coordinates in four cells (rows) for four genes (columns) with inferred weights for Factors 0, 1, 2 shown at the top of the columns. Pdia6 has high weight on Factor 2, Amotl2 on Factor 0 and 2, Ddb1 on Factor 0 and Kctd10 on Factor 0 and 1. (b) Visualization of three factors inferred in the same cells. Factor 0 is active around the cell center, Factor 1 at the cell border and Factor 2 in the cell center. (c) Hierarchical clustering of inferred gene weights recovers known gene clusters from Eng et al. (2019), indicated by label colors (purple: nucleus/perinucleus, cyan: cytoplasm, olive: protrusions). (d) Gene loadings for signatures identified in Mah et al. (2022) correlate strongly with inferred weights in (c), the displayed value is the Pearson correlation with the FISHFactor weights.

Lastly, we applied FISHFactor to a real dataset that comprises single-molecule data for 10 000 genes in 225 segmented cultured mouse embryonic fibroblasts (NIH/3T3) from a seqFISH+ experiment (Eng et al. 2019). As input for FISHFactor we used all cells and considered genes with a minimum of 30 molecules on average across cells, resulting in a total of 104 genes. Exemplary molecule coordinates for 4 genes in 4 cells are shown in Fig. 5a.

From these data, FISHFactor identified 3 factors capturing major subcellular expression patterns (Fig. 5b). The factors show distinct subcellular activities, with Factor 0 mainly being active around the cell center, Factor 1 at the cell border and Factor 2 inside the cell center. The inferred weight matrix (Fig. 5c) shows a clear clustering of genes into 3 clusters, where Factor 0 has high weights for genes previously annotated to cytoplasm (Eng et al. 2019) (Fig. 5c, cyan), Factor 1 for genes previously annotated to protrusions (Eng et al. 2019) (Fig. 5c, olive) and Factor 2 for genes previously annotated to nucleus/perinucleus (Eng et al. 2019) (Fig. 5c, purple). We compared this gene clustering with a clustering based on inferred weights of NMF applied to normalized and transformed gene counts per cell, and found that the clustering differs substantially, allowing the conclusion that the spatial information is needed to reconstruct the clusters in Eng et al. (2019) (Supplementary Fig. S5).

We asked to what extent the unsupervised approach of FISHFactor is able to recover gene loadings for signatures previously identified on this data based on manual annotation of genes to cellular regions (Mah et al. 2022). For this, we compared the factor weights inferred by FISHFactor to the previously identified signature loadings for the same set of genes (Fig. 5d) and found a strong correlation. This indicates that FISHFactor is able to retrieve the same information, but in a completely unsupervised manner, requiring only the number of latent factors as input. Moreover, the FISHFactor weights appear to be more sparse compared to the signature loadings from Mah et al. (2022), where loadings for signature 0 strongly correlate with loadings for signature 1 (Fig. 5d).

Overall, this application demonstrates that FISHFactor can reveal the major subcellular localization patterns in a data-driven manner without the need for manual labeling or segmentation of areas within the cell and identifies relevant gene clusters based on their subcellular colocalization.

Finally, we trained two FISHFactor models on the dataset: one trained on the first 25 cells (*complete*) and another trained on the first 20 cells (*incomplete*). We used Gaussian kernel density estimates of the molecule coordinates with the bandwidth determined by Scott’s rule (Scott 2015) to project the five hold out cells that were not considered in the *incomplete* model onto the latent factors using the weight matrix inferred using the *incomplete* model (Supplementary Fig. S6). The average correlation *R* = 0.72 between the projected factors (using *incomplete* model) and the inferred factors (using *complete* model) in the same cells indicates that projecting new data onto factors using a trained model is feasible but less accurate than training the model on the full dataset.

## 4 Discussion

The spatial localization of individual RNA molecules in a cell has long been limited to only a handful of genes at a time. However, recent technological developments have dramatically increased the number of genes that can be profiled, thereby enabling single-molecule resolution of spatial transcriptomics. This opens up the application of computational methods that share information across several genes, such as matrix factorization, which is based on the assumption that spatial expression densities of genes can be linearly decomposed into a small number of initially unknown patterns. While the benefits of such approaches have been demonstrated for spatial transcriptomics data on the cellular level (Velten et al. 2022, Townes and Engelhardt 2023), it was unclear how and whether similar ideas could be used to gain insights into the localization patterns of individual molecules at the subcellular level.

Here, we addressed this question by developing FISHFactor, a spatial non-negative factor model for single-molecule resolved spatial transcriptomics data that facilitates the identification of major subcellular expression patterns and co-localization of genes. We demonstrated that the use of a tailored likelihood model for single-molecule data based on a Poisson point process is beneficial compared to naive application of existing factor models that require data aggregation via binning. In addition to sharing information across all genes, FISHFactor furthermore enables sharing information across cells by jointly modeling expression patterns in hundreds of cells, which could improve the reconstruction accuracy of the weights and factors in our simulation studies and provides a direct means to compare expression patterns across cells. A joint modeling of cells can be particularly useful when the number of detected molecules per cell is relatively low and a single cell is not sufficient to reliably identify colocalization patterns of genes. We showed how FISHFactor scales with different numbers of molecules, factors, and cells, and demonstrated its applicability to datasets of 1000 cells. Moreover, we showed that the results are very stable for different random initializations. We demonstrated the value of FISHFactor for the unsupervised analysis of single molecule resolved data by an application to a dataset of cultured mouse embryonic fibroblasts, where the method identified relevant subcellular expression patterns and gene clusters based on subcellular spatial colocalization. Notably, these clusters cannot be detected on the cellular level, underlining the importance of considering subcellular information. In addition, we showed that it is in principle possible to project new data on latent factors if a reliable weight matrix has been inferred using a smaller number of cells. This could, for example, be useful to reduce training time on very large datasets or to incorporate new data into an existing model.

While the model is scalable to 100–1000 s of cells, a limiting factor for large-scale applications can be the linear scaling of memory allocation with the number of molecules per cell, which, for example, limits the application to ∼10 000 to 20 000 molecules per cell on a typical gpu with 24 GB of RAM. Future extensions of the model could address this by implementing subsampling strategies on the level of genes or molecules and developing approaches for an automated choice of relevant genes. While being a fully unsupervised method for detection of subcellular expression patterns, FISHFactor currently does not implement a method to determine the optimal number of latent factors and this needs to be chosen by the user. An appropriate choice can be guided by prior knowledge or heuristics as implemented by other factor models. For example, a Scree plot (Cattell 1966) based on a PCA with binned molecule coordinates could serve as an orientation. Importantly, the current implementation of FISHFactor relies on having accurate cell segmentations available to assign molecules to cells. For future research, it would therefore be interesting to investigate the benefits of joint segmentation and modeling approaches. Moreover, lifting the restriction of a single shared weight matrix for all cells and instead allowing a priori unknown groups of cells to share group-specific weight matrices would make the model even more flexible for heterogeneous cell populations with different gene co-localizations. Lastly, FISHFactor could also be extended to coordinates in 3D, where the same mathematical model can be used to enable an even broader applicability.

## Supplementary Material

btad183_Supplementary_Data

## Data Availability

The data used in this article were accessed from Zenodo, at https://doi.org/10.5281/zenodo.2669683; and from figshare, at https://doi.org/10.6084/m9.figshare.15109236.v2.
