# Supplementary material for: FISHFactor: a probabilistic factor model for spatial transcriptomics data with subcellular resolution
Source: Bioinformatics. 2023 Apr 11;39(5):btad183. doi: 10.1093/bioinformatics/btad183 (PMC10176502; doi:10.1093/bioinformatics/btad183)
Supplement: btad183_Supplementary_Data [file btad183_supplementary_data.pdf]

Supplementary Data

Florin C. Walter[1,2], Oliver Stegle[1,2,3], and Britta Velten[1,3]

[1]Division of Computational Genomics and System Genetics, German Cancer Research Center (DKFZ), Heidelberg, Germany
[2]Genome Biology Unit, European Molecular Biology Laboratory (EMBL), Heidelberg, Germany
[3]Cellular Genetics Programme, Wellcome Sanger Institute, Hinxton, Cambridge, United Kingdom

1 Supplementary figures

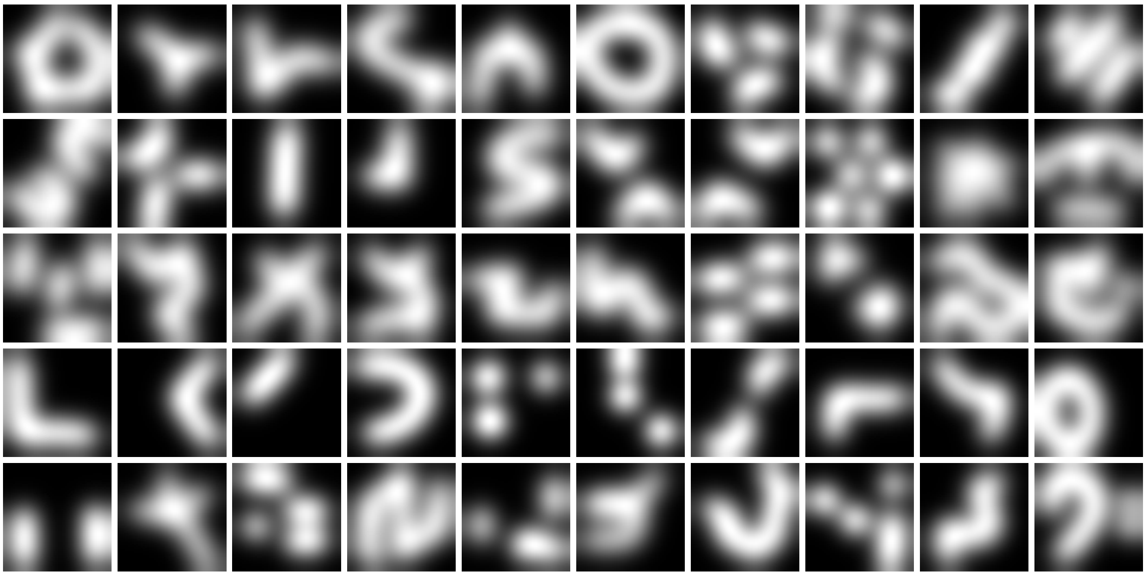

Figure S1: 50 hand painted factors used in the data simulation with intensity values from 0 (black) to 1 (white) and 50 x 50 pixels resolution. For every cell in the simulation, factors were randomly selected and random rotations of 0, 90, 180 or 270 degrees and random horizontal and vertical flips were applied.

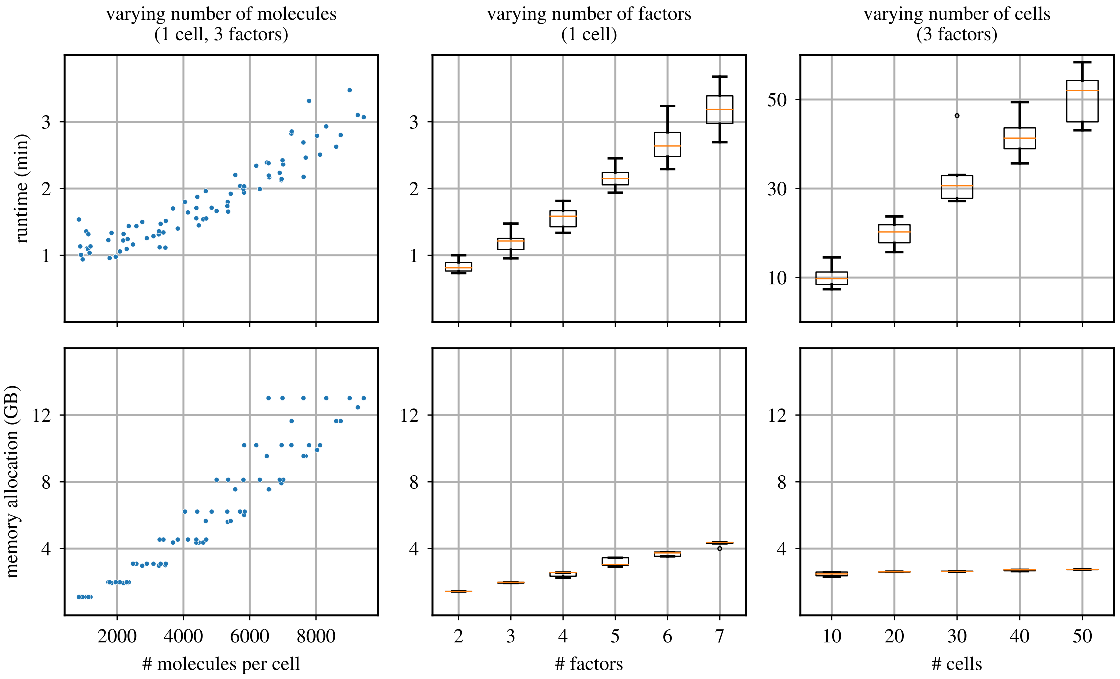

Figure S2: Scalability of FISHFactor. run time (first row) and maximum memory allocation (second row) are shown for scenarios with varying numbers of molecules per cell (first column), varying numbers of factors (second column) and varying numbers of jointly modeled cells (third column). The expected number of molecules per cell is kept constant in the second and third column. The run time scales approximately linearly in all cases, while the memory allocation scales linearly in the number of molecules per cell and the number of factors, but remains approximately constant in the number of cells. In scenarios with multiple cells, the memory allocation depends on the cell with the highest molecule count. This explains the slight increase of memory with increasing number of cells, because additional cells with higher molecule count are included.

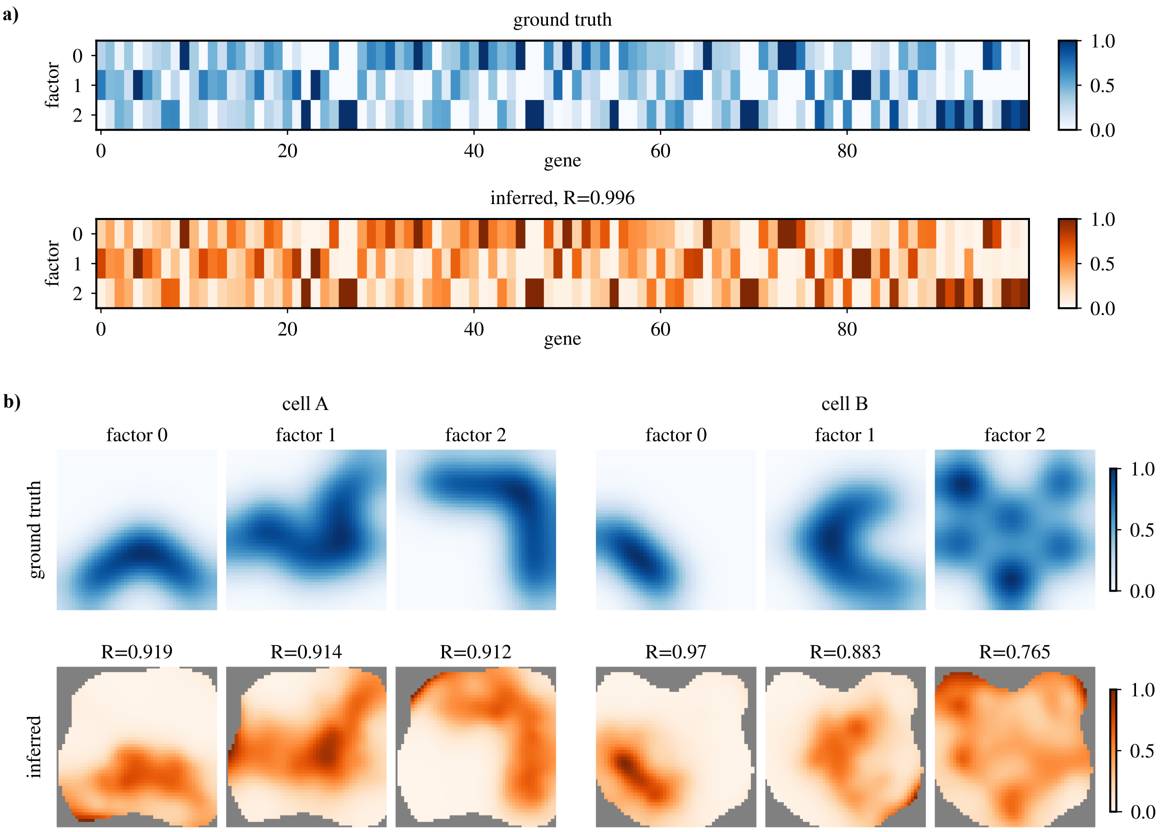

Figure S3: Application of FISHFactor to a large simulated data set with 1000 cells and 100 genes. **a)** The simulated (first row) and inferred (second row) weight matrices correlate strongly. **b)** Examples of simulated (first row) and inferred (second row) factors in two cells. R denotes the Pearson correlation coefficient.

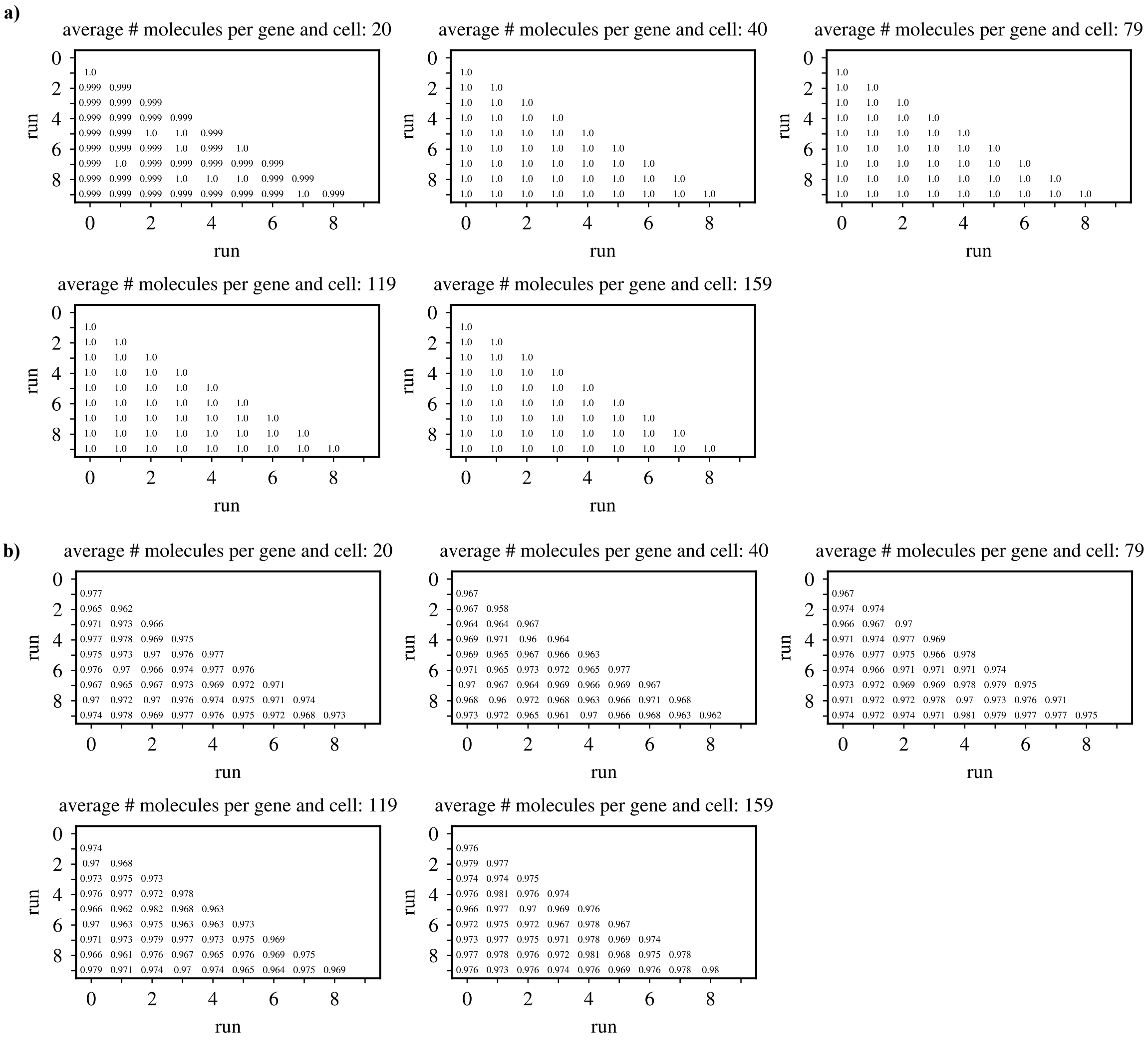

Figure S4: Reproducibility of inferred weights and factors in 5 simulated data sets (intensity scale factors $\mu_{dm} = 50, 100, 200, 300, 400$) with 20 cells with shared weight matrices each, considering 10 runs (with different random seeds) per data set. **a)** Pearson's correlation of inferred weights. **b)** Pearson's correlation of inferred factors.

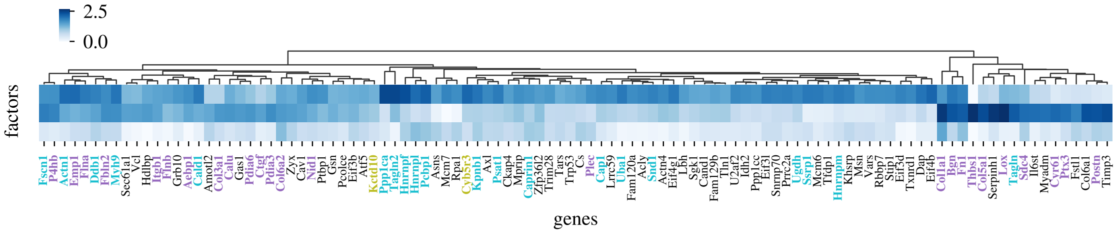

Figure S5: Gene clustering based on total RNA counts per cell, without including spatial information. Gene counts were normalized by dividing by the total count per cell (considering all 10 000 genes) and transformed as $\log(10000x + 1)$ where x is the normalized gene count. NMF with 3 components was performed with the normalized and transformed gene counts and genes were clustered using hierarchical clustering on the weights. Label colors denote clusters identified in Eng et al. (2019) (purple: nucleus/perinucleus, cyan: cytoplasm, olive: protrusions).

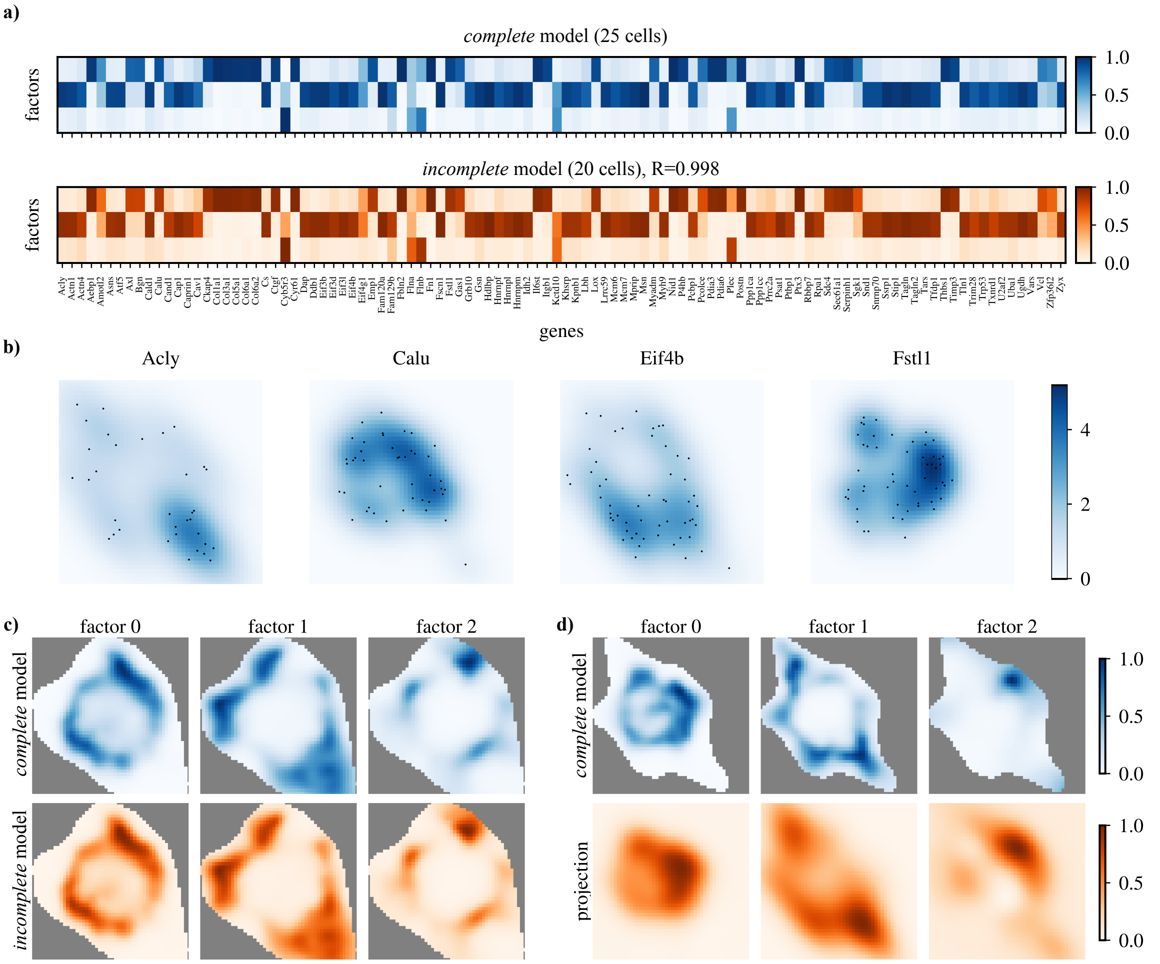

Figure S6: Projection of new data on factors using a trained model. **a)** First row: inferred weights using the first 25 cells of the 3T3 data set (*complete* model); Second row: inferred weights using the first 20 cells (*incomplete* model). **b)** Density estimates of four genes in a randomly selected cell using a Gaussian kernel density estimator. **c)** Inferred factors in a randomly selected cell from the *complete* model (first row) and the *incomplete* model (second row). **d)** Inferred factors in a randomly selected cell (that was included in the *complete* but not the *incomplete* model) from the *complete* model (first row) and projected factors using the density estimate and inferred weights from the *incomplete* model (second row).
